# Pilonidal Sinus in the Cheek: A Report of a Rare Case

**DOI:** 10.7759/cureus.80840

**Published:** 2025-03-19

**Authors:** Amer Al Ani, Mais Dawara, Riadh Bachir, Shahed A Dawara, Fawziya Al Rubaye

**Affiliations:** 1 General Surgery, Ajman University, Ajman, ARE; 2 Surgery, Dubai Medical College, Dubai, ARE; 3 Histopathology, Al-Bashir Teaching Hospital, Amman, JOR

**Keywords:** case report, cheek, pilonidal sinus, rare location, unusual site

## Abstract

Pilonidal sinus (PNS) is a common inflammatory condition that predominantly affects males. It typically occurs beneath the skin of the sacrococcygeal region. We present a rare case of a 40-year-old patient with a painless, suppurating swelling on the cheek for two days, accompanied by itching. Under local anesthesia, incision and drainage were performed, as the patient declined excision. Medical advice was provided, and the patient was encouraged to follow up. The cheek is an extremely rare location for a PNS. Diagnosis can be challenging due to its unusual presentation. However, special care should be taken during surgical excision, as preserving the cosmetic appearance is a priority.

## Introduction

Pilonidal sinus (PNS) is a common inflammatory condition affecting the subcutaneous fatty tissue. Over the past 50 years, its incidence has increased significantly [1], with reports estimating that it affects approximately 0.026% of the population [2]. PNS can present acutely or chronically, with the acute form being more common [3]. Despite ongoing research, its exact etiology remains unclear. Two main theories attempt to explain its development: the congenital theory, which suggests that a pre-existing subcutaneous sinus is present at birth and becomes infected later in life, and the more widely accepted acquired theory, which proposes that repeated friction, microtrauma, and pressure contribute to its formation [2]. Diagnosis is primarily clinical, requiring the exclusion of differential diagnoses, and histopathological analysis is performed when necessary to confirm the nature of the lesion.

## Case presentation

**Figure 1:**
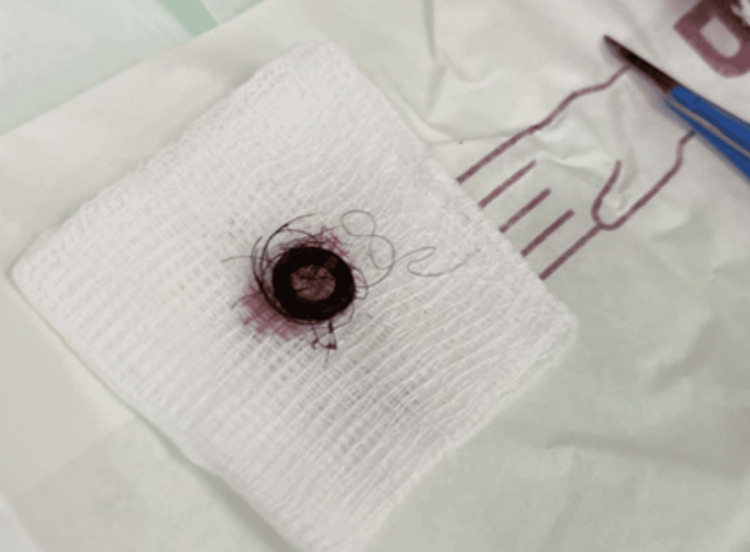
The excised tuft of hair from the pilonidal sinus.

A previously healthy 40-year-old Omani male presented in August 2023 with a two-day history of swelling in the left cheek. The onset was sudden and accompanied by itching. On examination, a localized bulge with an opening was noted, discharging yellow pus. Although the patient denied pain, tenderness was observed. He had no history of fever, chills, or medication use. After discussing treatment options, the patient declined an excisional biopsy. Consequently, an incision and drainage procedure was performed under local anesthesia, including curettage of the sinus and removal of an embedded tuft of hair (Figure 1).

**Figure 2:**
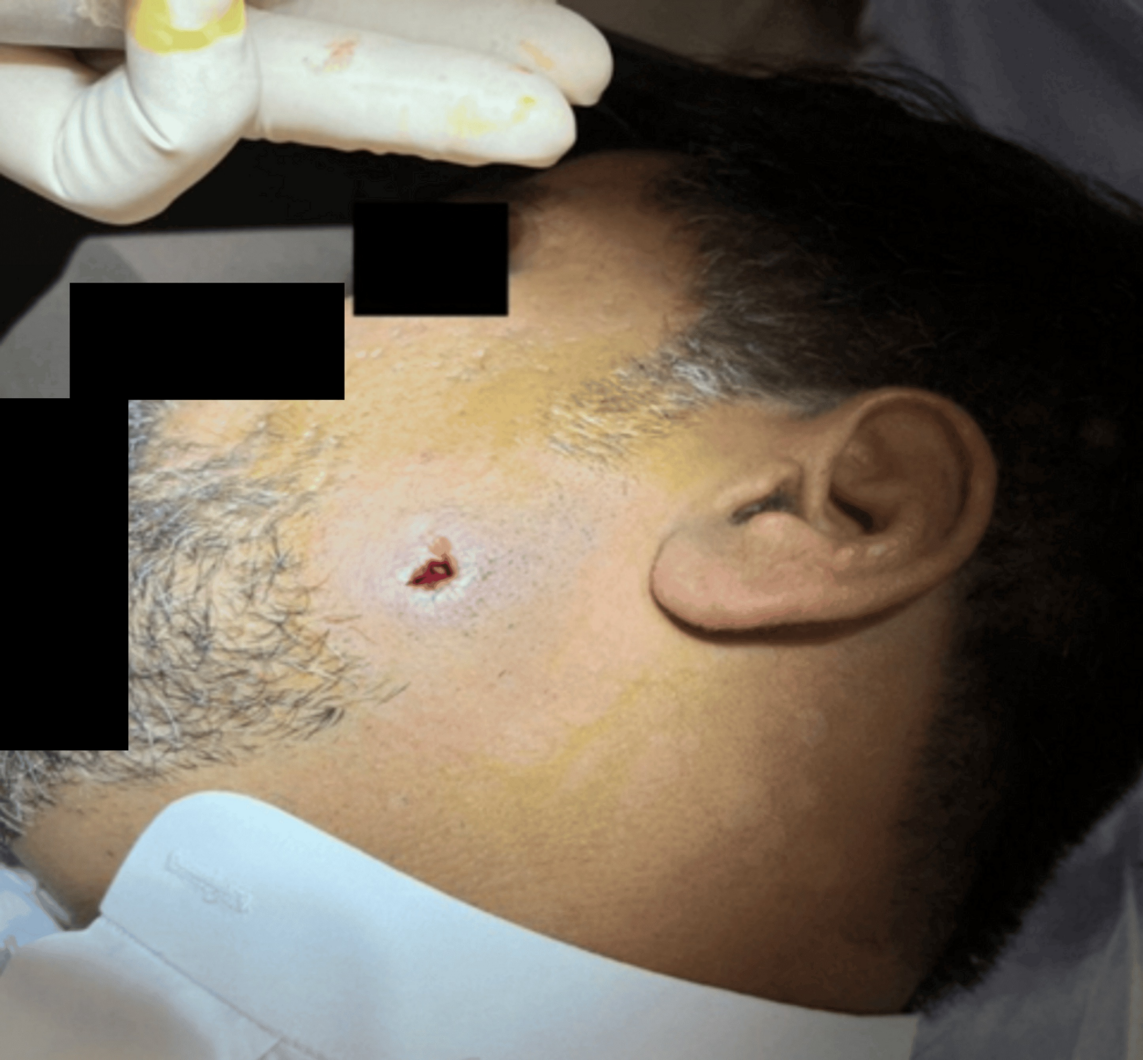
Pilonidal sinus wound on the left cheek left to heal by secondary intention after incision and drainage of the abscess with curettage of the sinus.

The wound was left to heal by secondary intention (Figure 2).

As no excision was performed, histopathological confirmation was not possible. The patient was advised on wound care, hygiene, and hair removal practices. At the three-month follow-up, the wound had healed completely, and the patient was satisfied with the outcome. He was encouraged to return if a recurrence occurred.

## Discussion

Historically, PNS was referred to as the Jeep disease due to its high prevalence among American soldiers who frequently drove jeeps during World War II [2]. The condition is more common in men [4] and rarely occurs before puberty or after the age of 40, likely due to hormonal influences on hair growth [5]. Risk factors include the presence of hair, deep skin creases (such as the intergluteal cleft), scarring, inadequate hygiene, and prolonged sitting or friction [6]. While sacrococcygeal PNS is the most common form, cases occurring in atypical locations, such as the face, are being more frequently reported [7]. A 2023 systematic review reported only seven cases of facial PNS, with the cheek being an extremely rare site, documented in just three cases [6,8,9].

Diagnosis of PNS is typically clinical, and imaging is generally unnecessary unless alternative conditions such as Crohn’s disease or neoplasia are suspected [3]. When excision is performed, histopathological analysis often reveals pseudo-cystic formations with granulomatous changes and hair debris. While the primary purpose of histological examination is to rule out malignancy, it also ensures complete excision with clear surgical margins.

There is no universally agreed-upon gold standard for PNS treatment [1]. Ideally, management should focus on removing sinus tracts, ensuring proper wound healing, and minimizing recurrence. Most cases require surgical intervention, but all surgical techniques have disadvantages, including poor wound healing, discomfort, long recovery periods, and potential recurrence [1]. Non-surgical options, such as pit picking, laser ablation, and phenol application, have been explored as alternatives [3]. A more recent approach, Salih’s preparation, involves injecting a solution into the sinus, regular wound care, shaving, and maintaining dryness. This method was tested on 400 patients, achieving an 89% cure rate with no recurrence [1].

The management of sacrococcygeal PNS differs from facial PNS, where surgical intervention is preferred for optimal cosmetic outcomes [6]. Conservative approaches have advantages such as fewer complications, shorter recovery time, and reduced discomfort, but their effectiveness in preventing recurrence remains controversial [1]. In asymptomatic cases or postoperative care, conservative management focuses on local hygiene, hair removal (e.g., laser epilation), and monitoring for infection. However, symptomatic patients generally require surgery. Acute PNS is treated with simple excision, whereas chronic or recurrent cases may necessitate radical resection with extended margins. The wound can then be managed through primary closure or secondary intention healing. Studies indicate that primary closure allows faster healing but carries a higher recurrence risk, while secondary intention reduces recurrence rates by 58% [4]. Negative pressure wound therapy has also been used to enhance healing [4]. The resultant defect can also be reconstructed using a flap, which allows for a wider resection, thereby reducing the risk of recurrence while simultaneously promoting faster healing. Additionally, flap reconstruction requires less intensive wound management compared to secondary intention healing [2].

In the case presented, the patient underwent incision and drainage with curettage, a decision based on his preference to avoid excision. However, limiting treatment to incision and drainage is not ideal, as it increases the risk of recurrence and prolongs healing time [2]. Furthermore, secondary intention healing in facial PNS can lead to undesirable scarring. The standard treatment for facial PNS is excision with primary closure under general or local anesthesia [6]. This differs from the management of PNS in other locations, where non-surgical options may be viable. Due to the cosmetic implications, facial PNS should be thoroughly resected to prevent recurrence, with primary closure preferred to minimize scarring. Facial skin is more flexible than other areas, making primary closure more effective and reducing the likelihood of visible grooves or creases. Unlike sacrococcygeal PNS, facial PNS generally does not recur if excised completely [6].

## Conclusions

Cheek PNS is an extremely rare condition and should be considered in the differential diagnosis of subcutaneous nodules. The definitive treatment is excision with primary closure to achieve the best cosmetic and functional outcomes. Managing facial PNS presents unique challenges, as preserving cosmetic appearance is a priority. This raises an important consideration regarding the role of conservative management in such cases.
